# The roles of FGFR3 and c-MYC in urothelial bladder cancer

**DOI:** 10.1007/s12672-024-01173-z

**Published:** 2024-07-20

**Authors:** Dereje E. Bogale

**Affiliations:** School of Medicine, Department of Oncology, Addis Ababa University, Addis Ababa, Ethiopia

**Keywords:** Urothelial bladder cancer, Fibroblast growth factor receptor 3, c-MYC

## Abstract

Bladder cancer is one of the most frequently occurring cancers worldwide. At diagnosis, 75% of urothelial bladder cancer cases have non-muscle invasive bladder cancer while 25% have muscle invasive or metastatic disease. Aberrantly activated fibroblast growth factor receptor (FGFR)-3 has been implicated in the pathogenesis of bladder cancer. Activating mutations of *FGFR3* are observed in around 70% of NMIBC cases and ~ 15% of MIBCs. Activated FGFR3 leads to ligand-independent receptor dimerization and activation of downstream signaling pathways that promote cell proliferation and survival. FGFR3 is an important therapeutic target in bladder cancer, and clinical studies have shown the benefit of FGFR inhibitors in a subset of bladder cancer patients. *c-MYC* is a well-known major driver of carcinogenesis and is one of the most commonly deregulated oncogenes identified in human cancers. Studies have shown that the antitumor effects of FGFR inhibition in FGFR3 dependent bladder cancer cells and other FGFR dependent cancers may be mediated through c-MYC, a key downstream effector of activated FGFR that is involved tumorigenesis. This review will summarize the current general understanding of FGFR signaling and MYC alterations in cancer, and the role of FGFR3 and MYC dysregulation in the pathogenesis of urothelial bladder cancer with the possible therapeutic implications.

## Introduction

Bladder cancer is the 10th most common cancer worldwide with an estimated 573,000 new cases and 213,000 deaths in 2020, and it accounts for 3% of all newly diagnosed cancers [1]. Urothelial carcinoma is the commonest histologic sub-type constituting around 90% of bladder cancer cases worldwide [2]. Urothelial bladder carcinoma (UBC) is generally classified as non-muscle invasive bladder cancer (NMIBC) and muscle invasive bladder cancer (MIBC). At diagnosis, 75% of UBCs are NMIBC, while 25% are MIBC or metastatic disease [3]. NMIBCs are treated with transurethral resection of bladder tumors (TURBT) and intravesical therapies [4, 5]. The standard of care for MIBC is radical cystectomy, cytotoxic chemotherapy and radiotherapy, while cisplatin-based chemotherapy remains the initial choice of therapy in the metastatic setting [6]. The median overall survival in patients with metastatic UBC treated with cisplatin-based regimens ranges from 13 to 16 months [7]. After the FDA (US Food and Drug Administration) approval of atezolizumab (PDL-1 inhibitor) in 2016 and the pan fibroblast growth factor receptor (FGFR) inhibitor erdafitinib in 2019, immune check point inhibitors (ICIs) and FGFR inhibitors are also included in the current armamentarium to treat locally advanced and metastatic UBC [8, 9].

Molecular studies have identified NMIBC and MIBC as patho-biologically distinct entities, and they appear to evolve through distinct molecular pathways [10]. NMIBCs, which are mostly low-grade papillary tumors that commonly recur, show constitutive activation of the RAS-MAPK pathway mainly through the activation of FGFR3, while MIBCs are generally characterized by alterations in the p53 and retinoblastoma (RB) pathways that normally regulate the cell cycle [10, 11]. The fibroblast growth factor receptor (FGFR) family consists of four transmembrane receptor proteins (FGFR1-4), and genomic alterations of these receptors have been documented in various types of cancers [12]. FGFR3 is frequently altered in UBC, and aberrant activation of FGFR3 has long been implicated in the pathogenesis of bladder cancer [13, 14]. Activating mutations of *FGFR3* are observed in around 70% of NMIBC cases and ~ 15% of MIBCs [14]. Activation of FGFR3 leads to constitutive ligand-independent dimerization of the receptor and activation of downstream signaling pathways that promote cell proliferation and survival [13].

The proto-oncogene *c-MYC* encodes the c-MYC (here after MYC) protein, a basic helix–loop–helix/leucine zipper (bHLHLZ) transcription factor that regulates the expression of many target genes and plays an important role in diverse cellular processes including cell growth and proliferation, differentiation, cellular metabolism, cell cycle progression, apoptosis, angiogenesis, and stem cell biology [15]. Due to its critical involvement in the regulation of many vital cellular processes, the expression of MYC as well as the levels of MYC mRNA and protein are tightly regulated at multiple levels to avoid tumorigenesis [16]. Deregulation of MYC that could occur due to transcriptional overexpression (as in gene amplification, translocation or altered upstream signaling) and/or protein stabilization, leads to high MYC protein levels that consequently drives tumor initiation, progression and maintenance [17]. Indeed, *MYC* is a well-known major driver of carcinogenesis and is one of the most commonly deregulated oncogenes identified in human cancers [18]. In UBC, MYC overexpression has been documented but the detailed mechanism of its dysregulation has not been described [19]. This review summarizes the general aspects of FGFR signaling and MYC alterations in tumorigenesis. Then it focuses on FGFR3 and MYC dysregulation in urothelial bladder cancer pathogenesis as well as on the potential therapeutic implications.

## FGFR structure and FGF-FGFR signaling

The FGFRs are a family of tyrosine kinases that constitute four different receptors: FGFR1–FGFR4, each consisting of three extracellular immunoglobulin (Ig) domains (I, II, and III), a transmembrane domain, and cytoplasmic tyrosine kinase domains [20]. The extracellular region has a hallmark serine rich sequence called the acid box in the linker region between the Ig-I and Ig-II domains, and it is thought that the first Ig domain (Ig-I) and the acid box are involved in receptor autoinhibition while the Ig-II and Ig-III domains are important for ligand (FGF) binding [20]. In addition to the different genes that encode the four FGFRs (FGFR1-4), alternative splicing of *FGFR* genes produces different FGFR isoforms that contribute further to the FGFR diversity. Alternative splicing of three exons (exons 7, 8 and 9) in the third Ig domain of FGFR1–FGFR3 gives rise to two different isoforms, FGFR(1–3)-IgIIIb (involving exons 7 & 8) and -IgIIIc isoforms (involving exons 7 & 9), which are predominantly expressed in epithelial and mesenchymal tissues respectively [21, 22]. In contrast, *FGFR4* lacks alternative exons encoding its Ig-III domain, and it has only the IgIIIc variant [23]. The binding specificity of FGFs to FGFRs differs amongst the -b (-IgIIIb) and -c (-IgIIIc) isoforms. While FGF1 is considered a universal FGF which can activate all FGFRs, the other FGFs favorably bind either to the -b or the -c FGFR isoforms [24, 25]. The FGF1/4/8/9/19 subfamilies (described next), which are mostly expressed in epithelial tissues (some of these FGFs like FGF4, 5, & 6 may also be expressed in mesenchymal tissues), favorably bind to the “c” isoforms, while the “b” isoforms show binding specificity for the mesenchymal tissue expressed FGF7 subfamily [24, 25].

Eighteen FGFs (FGF1-FGF10, FGF16-FGF23) have been described so far which can bind to the immunoglobulin domains (IgII & IgIII) of FGFRs to mediate their biological effects [26]. These FGFs which comprise the FGF family are categorized into six subfamilies: FGF1 (FGF1 and FGF2); FGF4 (FGF4, FGF5, and FGF6); FGF7 (FGF3, FGF7, FGF10, and FGF22); FGF8 (FGF8, FGF17, and FGF18); FGF9 (FGF9, FGF16, and FGF20); and FGF19 (FGF19, FGF21, and FGF23) [27]. FGFs also interact with the coreceptors of FGF/FGFR cascade which include heparan sulfate proteoglycans (HSPGs) (for paracrine FGFs) and the transmembrane protein Klotho (for endocrine FGFs) to stabilize binding to FGFRs [26, 28]. The first five subfamilies mentioned above are paracrine subfamilies, which have high affinity for heparan sulfate proteoglycans (HSPG) that causes them to act in a localized manner near the source of their expression [20, 27, 29]. By contrast, FGF19, FGF21 and FGF23 (FGF19 subfamily members) have decreased binding to HSPGs, allowing them to permeate through the HSPGs-rich extracellular matrix away from their source into the circulatory system to function in an endocrine manner [20, 26, 29]. Decreased affinity for HSPGs also reduces the ability of HSPGs to promote the binding of these FGFs to their receptors, making the endocrine FGFs dependent on the presence of members of the Klotho family (α-Klotho, β-Klotho, and Klotho-LPH related protein (KLPH)) in their respective target tissues for a stable FGF/FGFR binding in order to initiate cellular signaling [28, 29].

**Fig. 1:**
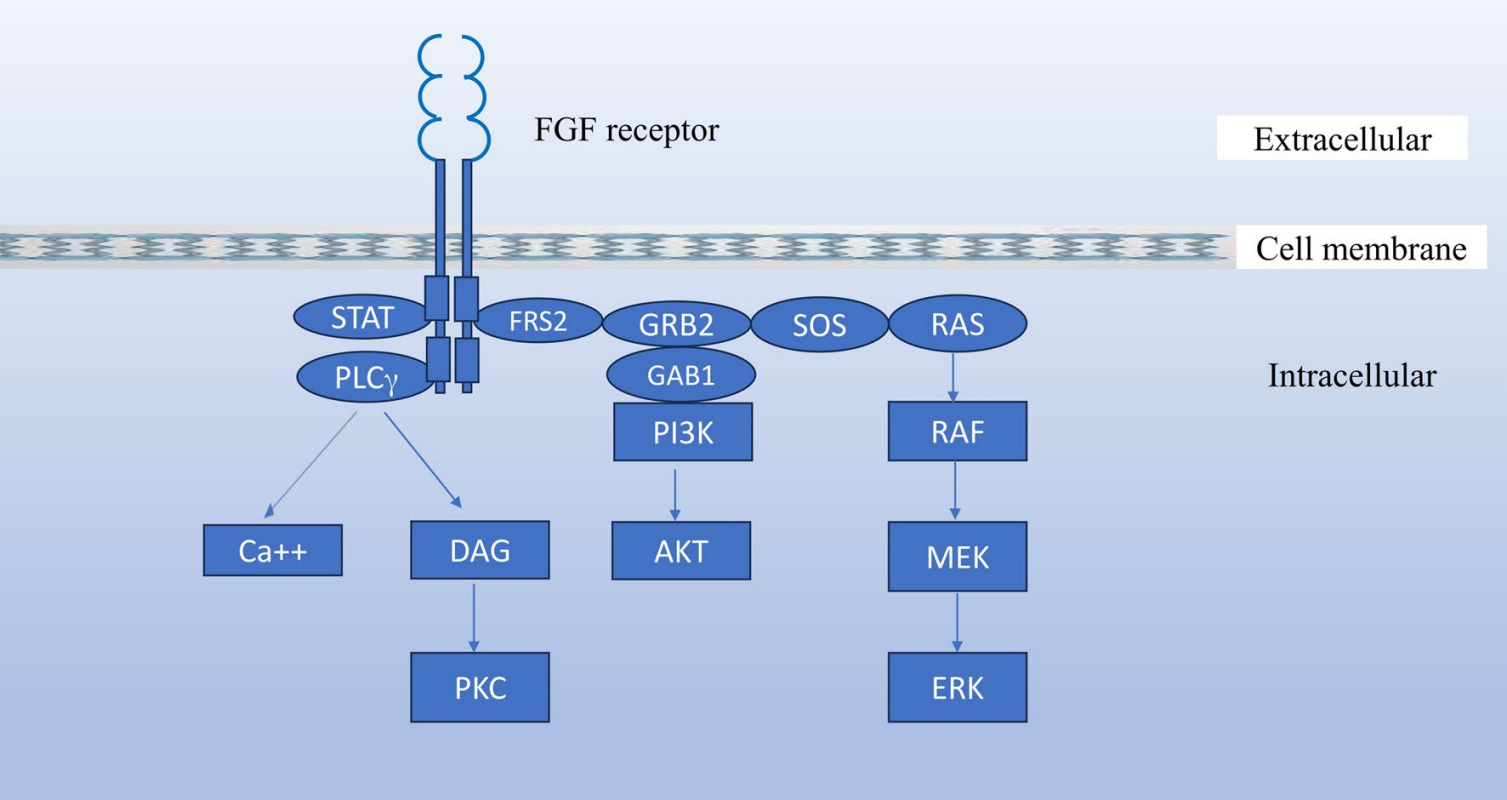
Signaling pathways activated by the FGFRs

FGF binding to FGFRs results in receptor dimerization, transphosphorylation, and activation of downstream signaling through four major pathways: RAS-MAPK, PI3K-AKT, Phospholipase C (PLC)γ, and signal transducers and activators of transcription (STAT) signaling pathways (Fig. 1) [8, 30]. Phosphorylation of specific tyrosine residues within the intracellular domain of the activated receptor tyrosine kinases (RTKs) results in recruitment of specific adaptor proteins that mediate the intracellular signaling. The small adaptor protein, growth factor receptor-bound 2 (Grb2), is known to link receptor tyrosine kinases with the RAS signaling pathway by binding to the guanine nucleotide-exchange factor SOS through its Src homology (SH) 3 domains, and to tyrosine-phosphorylated receptors or docking proteins via its SH2 domain [31, 32]. Unlike other RTKs like EGFR which bind to Grb2 directly, FGFRs do not directly bind to Grb2 upon FGF stimulation [32]. Rather, they signal through FGFR substrate 2 (FRS2) which constitutes a primary pathway for the activation of downstream RAS–MAPK and PI3K–AKT intracellular signaling [30, 32].

FRS2 proteins (FRS2α and FRS2β) are docking proteins that are anchored to the cell membrane through myristylation, and they contain phosphotyrosine-binding (PTB) domains in their amino terminal region and multiple tyrosine phosphorylation sites that serve as binding sites for Grb2 and for the SH2 domain containing protein tyrosine phosphatase, Shp2 in their C terminal tails [33]. FGFR activation results in phosphorylation (activation) of FRS2α which binds to Grb2 directly, and in addition, Grb2 is also recruited indirectly as FRS2α interacts with the tyrosine phosphatase Shp2, resulting in tyrosine phosphorylation of Shp2 and complex formation between Shp2 and Grb2 [33, 34]. Grb2 then activates the RAS-MAPK pathway by recruiting SOS, and it also recruits and phosphorylates Grb2-associated binding protein 1 (Gab1), which in turn recruits PI3K to activate the PI3K-AKT signaling [21, 35, 36]*.* Additionally, phosphorylation of tyrosine at the carboxy-terminal region of FGFR (Eg. Y766 in FGFR1) creates a binding site for the SH2 domain of PLCγ, leading to phosphorylation and activation of PLCγ [37]. FGFRs can also activate STAT and ribosomal protein S6 kinase 2 (RSK2) [21, 36].

FGF/FGFR signaling is subject to different negative regulatory mechanisms. Negative regulators that have been described include the sprouty proteins (Spry), Cbl proto-oncogene E3 ubiquitin protein ligase (CBL), similar expression to *fgf* genes (SEF) and MAPK phosphatases (MKP) [36, 38]. Sprouty (Spry) proteins, which were first identified as inhibitors of FGF-induced signaling in drosophila, are known to regulate RTK-driven signaling pathways [39, 40]. In humans, four Spry homologues (*Spry1-Spry4*) have been described, most of which are ubiquitously expressed [41, 42]. FGF signaling induces the expression of Spry, which in turn negatively feedbacks on FGF/FGFR/MAPK signaling [42]. They appear to regulate FGFR signaling at various levels and multiple mechanisms of action have been described [38]. Sprouty proteins interact with Grb2 and compete with SOS for Grb2 binding, thereby preventing membrane localization of SOS and inhibiting RAS activation [43]. Sprouty may also regulate RAS signaling by acting at the level of RAF and inhibiting its activation [44, 45].

Another common mechanism by which RTK signaling is downregulated is through the removal of receptors from the plasma membrane via endocytosis following which, receptors are either recycled back to the cell surface or degraded in lysosomes [46]. Receptor ubiquitination is an important step in the endocytosis and subsequent degradation of receptors, and many reports have implicated the CBL (Casitas B-lineage Lymphoma proto-oncogene) family of E3 ligases in FGFR ubiquitination that leads to receptor degradation [38]. Following FGFR activation, it has been shown that Grb2 bound to FRS2α forms a complex with CBL resulting in the ubiquitination and degradation of FGFR and FRS2α, and attenuation of FGF/FGFR signaling [47].

MAPK phosphatases (MKP) are members of the dual specificity phosphatase (DUSP) family that regulate the MAPK pathway by dephosphorylating both threonine and tyrosine residues of activated MAP kinases [48]. FGFR signaling induces the expression of *DUSP6* which encodes MKP3, an ERK-specific MAPK phosphatase that regulates FGFR signaling by directly dephosphorylating MAPK (ERK1 and ERK2) on phosphor-tyrosine and phosphor-threonine residues [35, 38, 49]. FGF signaling also induces the expression of Sef (Similar expression to *fgf*), a transmembrane protein which functions as a feedback inhibitor of FGF signaling by blocking the activation of MAPK primarily, and also by inhibiting AKT activation [30, 50, 51]. In addition to the regulators discussed above, another protein that might modulate FGFR signaling is FGFRL1 (FGFR5), an atypical receptor that lacks an intracellular kinase domain and which cannot signal by trans-autophosphorylation [52]. FGFRL1 may negatively regulate FGF signaling, and among the proposed mechanisms include binding to FGF ligands and sequestering them by acting as a decoy receptor; or by forming heterodimers with other members of the FGFR family and thereby preventing trans-autophosphorylation as the dimeric complex would have only one tyrosine kinase domain [52].

The FGF-FGFR signaling is involved in diverse functions including development, proliferation, differentiation, survival, and tumorigenesis. The specific biological response to FGFR activation occurs in a context dependent manner, and a number of factors including the cell type, nature of activating FGFs, type of receptor involved, and the downstream pathways activated determine the eventual response [53].

## FGFR3 alterations in urothelial bladder *cancer*

Genomic alterations of FGFRs have been documented in various types of cancers and around 7% of all cancers harbor FGFR alterations [12, 54]. In bladder cancer, FGFR1 and FGFR3 alterations are commonly found and they have been implicated in the pathogenesis of UBC [55, 56]. In contrast, alterations involving FGFR2 are rare and their role in bladder carcinogenesis is minor, while FGFR4 alterations are hardly reported in UBC [56–58].

FGFR1 is the most commonly altered FGFR in human cancers [12, 54] but studies on FGFR1 in bladder cancer are very few. In one study, among 126 urothelial cancers (90 of them were UBCs), 7% were found to have *FGFR1* amplifications [54], while in a recent study of 4035 UBCs, *FGFR1* genetic alterations were detected in 3.7% of the tumors [56]. Increased expression of FGFR1 has also been demonstrated in a high proportion of UBC cell lines and tumors independent of stage and grade, which is thought to promote cell proliferation and survival via activation of the MAPK pathway [59]. In normal urothelial cells, two splice variants of FGFR1 IIIc, FGFR1α and FGFR1β, (FGFR1 β lacks exon 3 of FGFR1 α, removing the first Ig loop of the extracellular domain) are found to be expressed at similar levels [60]. In UBC, on the other hand, FGFR1β is expressed at higher levels, and the FGFR1β:FGFR1α expression ratio is significantly increased with increasing tumor stage and grade [60]. As FGFR1β has higher affinity for FGF1, it was suggested that this isoform switch may give a proliferative advantage that could play a role during tumor progression [60]. Additionally, it was indicated that FGFR1 could play a crucial role in invasion and metastasis, but its role in driving bladder cancer cell proliferation might be less important than FGFR3 [61].

*FGFR3* activating mutations, which frequently occur due to point mutations or gene fusions, are among the most common genetic abnormalities in UBC [55, 62]. They have been reported to occur in around 70% of NMIBCs and ~ 15 of MIBCs [14, 62, 63]. More than 10 different *FGFR3* missense mutations involving exons 7, 10 & 15 have been described [14, 55, 64]. Among these, R248C, S249C and Y375C account for more than 85% of the mutations, with S249C being the most common mutation contributing up to ~ 70% alone [14, 55, 63, 64]. These *FGFR3* mutations such as S249C, R248C and Y375C, that substitute wild-type residue with cysteine, allow for disulfide bridge formation between adjacent receptor molecules leading to constitutive receptor dimerization and subsequent receptor activation [57, 65, 66]. Other infrequently occurring mutations including the transmembrane domain mutations G382R and A393E have also been reported to promote receptor dimerization and ligand independent receptor activation [66–68].

*FGFR3* translocations that result in formation of oncogenic fusion proteins are found in 3–6% of bladder cancers [69]. Translocations of *FGFR3* with two distinct fusion partners, leading to the production of FGFR3-TACC3 and FGFR3-BAIAP2L1 fusion proteins have been well described, with *FGFR3-TACC3* fusions being the most common which were observed in 10 (2.4%) of 412 patients in The Cancer Genome Atlas (TCGA) muscle-invasive bladder cancer (MIBC) cohort [62, 70, 71]. These fusion proteins comprise exons 1–18 (amino acids 1–760) of FGFR3 fused in frame to different C-terminal regions of partner proteins [72]. It has been proposed that the FGFR fusion partners (TACC3 and BAIAP2L1) mediate oligomerization of the receptor, which triggers activation of the respective FGFR fusion proteins [73]. Consequently, the coiled-coil domain of TACC3 and the BAR (Bin-Amphiphysin-Rvs) domain of BAIAP2L1, which are predicted to induce dimerization, are retained almost intact in FGFR3-TACC3 and FGFR3-BAIAP2L1 fusion proteins respectively [72–74]. Transforming acid coiled-coil containing protein 3 (TACC3) is a member of the TACC family of proteins (TACC1, TACC2 and TACC3) that plays an important role in microtubule organization during mitosis [75]. Formation of FGFR3–TACC3 fusion protein constitutively activates the MAPK pathway, but not the PLCγ pathway due to loss of the tyrosine residue responsible for interaction with PLCγ (Y762) located in exon 19 of the *FGFR3* gene [72, 76, 77]. The second FGFR3 fusion partner, Brain-specific angiogenesis inhibitor 1-associated protein 2-like 1 (BAIAP2L1), along with BAIAP2 and BAIAP2L2, belong to the IRSp53 family of proteins [78]. The FGFR3–BAIAP2L1 fusion protein that dimerizes through the BAR domain results in constitutive FGFR3 kinase domain activation and promotes the MAPK signaling and STAT1 phosphorylation [74].

In addition to activating point mutations and fusions, wild type FGFR3 can contribute to tumorigenesis in bladder cancer. In normal urothelium, FGFR3 is the most abundantly expressed FGFR and two main isoforms of FGFR3 have been identified; FGFR3b, a full-length receptor, and FGFR3 Δ8-10, a soluble isoform lacking exons 8–10 which encode the third Ig domain and the transmembrane domain, and that can act as a dominant negative regulator of FGF induced proliferation [79]. In UBC, altered expression of FGFR3 isoforms results in a relative decrease in FGFR3 Δ8-10 expression and an isoform switch from FGFR3b to FGFR3c—another full-length FGFR3 isoform that is not detected in normal urothelial cells but binds to a wider range of FGF ligands [79]. Hence, wild type FGFR3 can contribute to UBC tumorigenesis through differential splicing that generates splice variants with altered ligand specificity, in addition to ligand independent receptor activation through dimerization of an overexpressed protein [80]. FGFR3 overexpression is common in UBC, and it is a major mechanism of FGFR activation in tumors with wild type FGFR3 [80, 81]. Overexpression is more frequent in tumors of low stage and grade, and high expression of FGFR3 is correlated with the presence of mutated *FGFR3* [80, 82, 83]. In non-invasive tumors, where activating *FGFR3* point mutations are very common, FGFR3 overexpression is found in up to 80% [72, 84]. In T1 tumors, on the other hand, overexpression has been documented in 40–70%, and in MIBC, around 40% of these tumors show FGFR3 overexpression [72, 80, 83–86].

The mechanism of FGFR3 overexpression in bladder cancer is not completely clear, but dysregulation of FGFR3 expression due to various alterations including microRNAs (miRs) and transcription factors have been described. Expression of miRs-99a/100 which target FGFR3 has been found to be inversely correlated with FGFR3 mRNA levels in bladder cancer [87]. Low grade tumors have been characterized by down regulation of many microRNAs including miRs-99a/100, and this downregulation of miRs-99a/100 may contribute to the upregulation of FGFR3 in NMIBCs [87]. In bladder cancer cell lines, it has been shown that FGFR3 expression is induced by hypoxia [88]. Hypoxia is a key feature of many solid tumors that contributes to tumor progression, invasiveness, metastasis and resistance to both chemotherapy and radiotherapy [89]. Hypoxia-inducible factor-1α (HIF-1α) accumulates in response to cellular hypoxia, and it is a critical regulatory protein of cellular response to hypoxia. The increased expression of FGFR3 in hypoxia is primarily dependent on HIF-1α but it is also partly dependent on miR-100, whose expression is suppressed by hypoxia independently of HIF-1α, and this may serve to augment the increased FGFR3 levels [88]. Other transcription factors implicated in the regulation of FGFR3 expression include the p53 family members, p63 and p73, that have been shown to induce *FGFR3* transcription [90]. MYC is also a direct positive regulator of FGFR3 expression at the transcriptional level and the FGFR3/MYC positive feedback loop has been described (will be discussed later) [91]. Finally, *FGFR3* gene amplifications which may result in upregulated expression are rare in UBC despite the reportedly high frequency of FGFR3 overexpression in this cancer [83, 92].

## FGFR3 signaling in urothelial bladder *cancer*

Despite the high frequency of FGFR3 activation and the known central importance of FGFR3 signaling in UBC tumorigenesis, studies that have investigated the downstream pathways mediated by FGFR3 activation and the subsequent cellular changes specifically in malignant urothelial cells are relatively few.

In immortalized normal human urothelial cells (TERT-NHUC), expression of mutant *FGFR3* (S249C, Y375C and K652E) induced activation of ERK1/2 and PLCɣ signaling, but not activation of AKT or SRC [93]. In contrast, in NIH-3T3, expression of similar mutant *FGFR3* types resulted in activation of ERK1/2, AKT, PLCɣ and SRC, and it also induced morphological transformation, cell proliferation, and anchorage independent growth [93]. In addition, STAT1 phosphorylation have not been detected in normal urothelial cells expressing mutant *FGFR3*, and PLCγ phosphorylation was only observed in TERT-NHUC expressing the common S249C and Y375C mutations, but not in the rare K652E mutation [93]. In UBC, activation of FGFR3 has been reported to activate the RAS-MAPK and possibly the PI3K/AKT pathway [66, 72, 91, 94]. Studies involving FGFR3 activated bladder cancer cell lines show major inhibition of p-ERK but not p-AKT following FGFR inhibitor treatment, which suggests that the MAPK pathway could be the major downstream signaling pathway that is activated [69, 95, 96]. This may also be further supported by the fact that *FGFR3* and *HRAS* mutations which result in MAPK pathway activation are mutually exclusive [97]. The activation of the PI3K/AKT pathway has been shown across the entire spectrum of UBCs regardless of FGFR3 status, suggesting that this pathway can be activated by several mechanisms [91, 98]. In FGFR3 activated UBCs, few studies have indicated the downstream involvement of the PI3K/AKT pathway. In a study by Gust et al. FGF-1 ligand stimulation of UM-UC1 urothelial cancer cell lines, which express a high level of wild-type *FGFR3*, resulted in activation of both ERK 1/2 and AKT [96]. In this study, inhibition of FGFR3 with R3Mab in the same cell lines abrogated receptor phosphorylation as well as phosphorylation of ERK1/2, but the effect on p-AKT level was not reported. In another study, the cell line 97–7, which carried the FGFR3 S249C mutation without additional mutations in *RAS, AKT* or *PIK3CA*, exhibited increased p-AKT levels compared with 5637 (FGFR3WT) and T24 (FGFR3WT) cell lines, and the increase was reversed by knockdown of FGFR3 indicating that the FGFR3 S249C mutation may activate the AKT signaling pathway [99].

Specific downstream effectors of activated FGFR3 signaling in bladder cancer have also been described. MYC has been reported to be a master regulator of proliferation that is activated downstream from FGFR3 (will be discussed later) [91]. The transcription factor ETV5 is also a downstream target of activated FGFR3 that is upregulated through the MAPK/ERK signaling pathway [100]. ETV5 has been shown to be involved in the crosstalk between FGFR3 signaling and the Hippo pathway; upregulates the expression of genes involved in EMT; and mediates proliferation and anchorage independent growth of bladder cancer cells [100].

## c-MYC and *cancer*

**Fig. 2:**
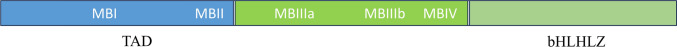
The structure of c-MYC protein. Location of MYC or M boxes (MB) I and II within the transactivation domain (TAD), and MBIII and IV in the central region of the protein. *bHLHLZ* basic helix-loop-helix leucine zipper

The MYC family includes MYC (c-MYC), MYCN (N-MYC), and MYCL (L-MYC) all of which belong to the superfamily of basic helix-loop-helix leucine zipper (bHLHLZ) DNA binding proteins [101]. MYC, the prototype member of the MYC family, is encoded by the *MYC* proto-oncogene located on human chromosome 8, which is among the most commonly deregulated genes during tumorigenesis occurring in over 70% of human cancers [101–104]. The MYC protein consists of an N-terminal region containing the transactivation domain, a central region involved in nuclear localization, and a C-terminal region which comprises a basic HLHLZ domain involved in interaction with its obligate partner, MAX and binding to DNA [101, 103]. Multiple highly conserved sequences known as MYC or M boxes (MB I, II, III and IV) are also located within the amino-terminal and central region that contribute to MYC function through interactions with different partners (Fig. 2) [105, 106]. MBI and MBII are located the within the transactivation domain (TAD) while the other M boxes are found in the central region of the protein [106]. Heterodimerization of MYC with its obligate partner MAX enables the MYC:MAX heterodimer to bind E-box DNA sequences in the regulatory regions of target genes and regulate expression [107].

MYC is estimated to regulate the expression of around 15% of human genes [108]. It regulates multiple cellular processes including cell growth, cell cycle, differentiation, apoptosis, angiogenesis, metabolism, ribosome biogenesis, protein synthesis, DNA repair, immune response, and stem cell formation [101, 108–110]. The specific effects of MYC activation depend on the cellular levels of MYC protein and cellular context [111]. It initiates tumorigenesis in a permissive genetic/epigenetic context & contributes to many of the hallmarks of cancer, including proliferation, self-renewal, cell survival, genomic instability, metabolism, invasiveness, angiogenesis and immune evasion [111, 112].

A tightly controlled transcription of the *MYC* proto-oncogene as well as abundance of MYC mRNA and protein is important to avoid its tumorigenic effect in normal cells [113]. In cancer, on the other hand, MYC is frequently deregulated through different mechanisms including aberrant signal transduction leading to increased MYC transcription or increased MYC mRNA and protein stability, amplifications, chromosomal translocations, and altered enhancer activity [114, 115]. Through these mechanisms, the level of MYC increases contributing to its potent role in both the initiation and maintenance of a tumorigenic state [114, 115].

Multiple aberrant signaling pathways are implicated in MYC deregulation. Activation of MAPK and PI3K/AKT signaling pathways, both of which are commonly dysregulated in cancer, can lead to MYC deregulation by altering MYC protein stability and accumulation [114, 116]. The two N-terminal phosphorylation sites in MYC, Thr-58 and Ser-62, are involved in the regulation of MYC protein stability in cells. Phosphorylation of Ser-62 mediated through the action of ERK results in the stabilization of MYC protein, while phosphorylation of Thr-58, which is dependent on prior Ser-62 phosphorylation and which is mediated by glycogen synthase kinase (GSK3), promotes MYC degradation through the ubiquitin/proteasome pathway [116]. AKT, an effector kinase of the PI3K/AKT pathway, phosphorylates and inactivates GSK3 thereby inhibiting the ability of GSK3 to phosphorylate and negatively regulate MYC protein stability [116, 117]. Both the PI3K/AKT and MAPK pathways can also indirectly regulate MYC through MAD1, a member of the MAD protein family (MAD1, MXI1, MAD3 and MAD4) which compete with MYC for binding to its obligate partner MAX [118]. Ribosomal S6 kinase (RSK) which is activated by ERK, and S6K1 which is a downstream effector of PI3K/AKT/mTOR pathway, have been shown to phosphorylate MAD1 that promotes its ubiquitylation and degradation, thereby allowing MYC to bind to MAX unhindered to promote proliferation and cellular transformation [119].

Other signaling pathways implicated in the regulation of MYC include WNT/β-Catenin, NOTCH and transforming growth factor-β (TGF-β) signaling pathways. Activation of the canonical WNT/β-catenin signaling pathway results in stabilization and accumulation of β-catenin which translocate into the nucleus leading to expression of WNT/β-catenin target genes, among which MYC is a well-established one [120]. NOTCH signaling is an important oncogenic pathway in T-cell acute lymphoblastic leukemia (T-ALL), and NOTCH1 directly activates MYC expression via NMe” (for NOTCH MYC enhancer), a long-range *MYC* enhancer located 1.4 Mb downstream of the *MYC* locus [115, 121]. Transforming growth factor β (TGF-β) signaling is another important pathway that negatively regulates MYC expression by forming a repressive complex in the promotor region of *MYC* [122, 123].

The MYC protein has a short half-life of around 30 min, and its stability & activity is also regulated by multiple other mechanisms [124]. One of the most important mechanisms to control MYC levels involves degradation via the ubiquitin–proteasome system, and many E3 ubiquitin ligases have been shown to interact with MYC to enhance its degradation or stabilize it [125]. Among these, two F-box proteins, Fbw7 and Skp2, that target MB1 and MB2 domains of MYC respectively have been well characterized [126]. Fbw7 (F-box and WD repeat domain-containing protein 7) is a member of the F-box protein family, which is part of the Skp1-Cullin-F-box-protein complex (SCF), an E3-ubiquitin ligase that ubiquitinates proteins and triggers proteasome degradation [126, 127]. MYC is a direct target of Fbw7 mediated ubiquitination that triggers proteasomal degradation, and this regulation of MYC by Fbw7 is dependent on MYC phosphorylation [126]. The phosphorylation of MYC at Ser-62 by ERK or by other kinases like cyclin dependent kinase 1 (CDK1) and the subsequent phosphorylation of Thr-58 by GSK3 is followed by dephosphorylation of Ser-62 by protein phosphatase 2A (PP2A), leaving the singly phosphorylated MYC at Thr-58 that is ubiquitylated by Fbw7 which promotes its degradation [114, 124, 128]. Skp2 (S-phase Kinase-Associated Protein 2), on the other hand, binds to MYC via its MB2 and HLH-Zip domains and not only mediates its ubiquitylation & degradation but it is also a potent stimulator of MYC transcriptional activity; and importantly, Skp2-mediated degradation of MYC is not dependent on the phosphorylation of Thr-58 [126, 129]. Many other additional proteins are also involved in the regulation of MYC protein stability including ubiquitin ligases that directly ubiquitinate MYC like, TRUSS, β-TrCP, TRIM32 & Fbx29; deubiquitinating enzymes that cleave ubiquitin chains to antagonize the activity of ubiquitin ligases like Usp28 & Usp36; and those that indirectly regulate MYC stability like SIRT2 & NEMO (NF-kB essential modulator) [125]. Of note, ubiquitination of MYC not only controls MYC protein levels, but also controls MYC transcriptional activity, and E3 ligases that destabilize MYC can either inhibit MYC activity or increase it. For example, Fbw7-mediated ubiquitination triggers proteasomal degradation and inhibits MYC activity, while Skp2 promotes MYC ubiquitination & degradation but also increases the transcriptional activity of MYC [125, 129].

Direct alterations involving the *MYC* gene such as amplifications or chromosomal translocations are another common mechanism of MYC deregulation. *MYC* amplification was found in 21% of the samples in a pan-cancer analysis of genomic and expression data of the TCGA dataset involving ~ 9000 samples covering 33 tumor types [130]. Constitutively high levels of MYC expression can result from translocations that juxtapose the *MYC* gene locus with other enhancer regions such as the immunoglobulin (Ig) heavy chain enhancer as in Burkitt lymphoma, or through complex chromosomal rearrangements as in the case of multiple myeloma [131, 132]. Lastly, altered enhancer activity has also been shown as a mechanism of MYC deregulation at its endogenous genomic locus [114, 115].

## c-MYC dysregulation in urothelial bladder *cancer*

**Fig. 3:**
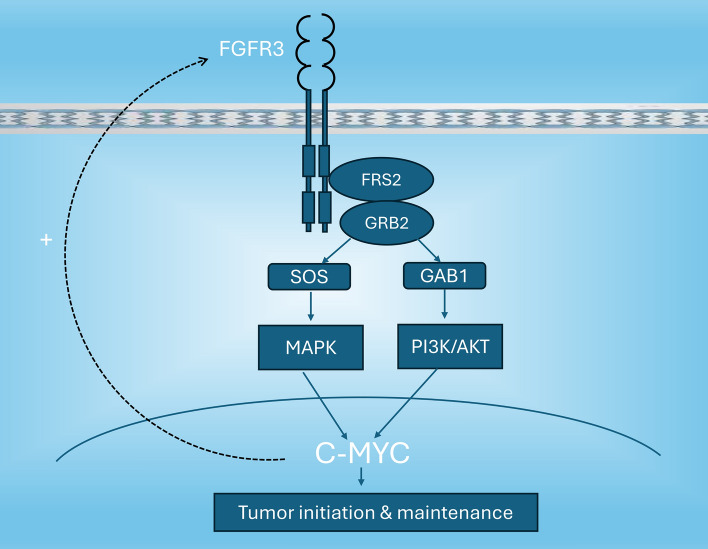
An illustration of c-MYC as a key downstream effector of activated FGFR3 mediating tumorigenicity in urothelial bladder cancer. The direct positive regulation of c-MYC on FGFR3 expression is also shown (dashed arrow)

MYC overexpression is commonly observed in many human cancers including bladder cancer [19, 133–135]. While the mechanism of MYC overexpression in UBC is not fully elucidated, MYC alterations due to dysregulated signaling have been described. The MYC transcription factor has been shown to be a key downstream effector of FGFR signaling, mediating tumorigenicity in different FGFR aberrant cancer cells including bladder cancer [136]. In a study with cell lines derived from urothelial bladder cancer (MGH-U3 (Y375C FGFR3 mutation) and RT112 (FGFR3-TACC3 fusion)), upregulated MYC expression due to constitutively activated FGFR3 has been described and, in addition, MYC has also been shown to be a direct positive regulator of FGFR3 expression at the transcriptional level forming an FGFR3/MYC positive feedback loop [91]. This finding was further supported by analysis of transcriptomic datasets of bladder tumors that showed significant MYC (mRNA) overexpression in bladder tumors harboring FGFR3 mutations, with MYC and FGFR3 expression levels being positively correlated, while in tumors bearing wildtype FGFR3, neither MYC overexpression nor such correlation was observed [91]. As a result of FGFR3 activation, both an increase in MYC mRNA levels due to activation of the p38α MAP kinase and stabilization of the MYC protein mainly due to activation of AKT were reported, and the authors emphasized the vital role of these two pathways in MYC accumulation in bladder cancer with aberrantly activated FGFR3 [91]. However, the role of ERK pathway in MYC protein accumulation was not investigated in the above study. In contrast, other studies involving cell lines of bladder cancer and other cancers have described the predominant role of ERK pathway in regulating MYC protein levels. In a study involving cell lines harboring FGFR1, FGFR2 and FGFR3 alterations, which included bladder cancer cell lines (UMUC14 & RT112), ectopic expression of undegradable mutant *MYC* conferred resistance to FGFR inhibition while FGFR inhibitor treatment reduced ectopically expressed wild type *MYC*, suggesting the important role of FGFR activation in maintaining MYC stability and blockage of FGFR signaling causes downregulation of MYC mainly due to protein degradation [136]. In this study, inhibition of the ERK pathway induced MYC protein level reduction, while the MYC protein level was not affected by AKT inhibition as well as by siRNAs against STAT3 or PLCɣ, suggesting that MYC protein stability is predominantly regulated by the FGFR-MEK-ERK signaling in FGFR aberrant cancers [136]. Based on the notion that ERK mediated phosphorylation of MYC Ser-62 results in stabilization of the MYC protein, and activated AKT phosphorylates and inactivates GSK3, thereby inhibiting the phosphorylation of MYC at Thr-58 that promotes MYC degradation, it is possible that both the ERK and AKT pathways may be involved in the regulation of MYC protein stability in bladder cancer with aberrant FGFR3 activation (Fig. 3) [116, 125, 126, 128]. However, considering the possibility that the ERK pathway may be the major downstream signaling pathway activated downstream of FGFR3 as discussed in the previous section, this pathway may play a more dominant role in inducing MYC protein accumulation in bladder cancer with aberrantly activated FGFR3.

Several proteins and RNAs that directly or indirectly interact with MYC affecting its expression and protein stability may also play roles in the pathogenesis of bladder cancer. POLD1, one of the four subunits of DNA polymerase δ (POLD1, POLD2, POLD3, and POLD4) and which is upregulated in many tumors including bladder cancer, has been shown to directly bind to MB1 domain of MYC competitively with Fbw7, preventing Fbw7-mediated MYC ubiquitination and degradation [137]. The subsequently elevated levels of MYC increases the transcription of POLD1 forming a positive feedback loop, and it was suggested that this non-enzymatic function of POLD1 may play a role in the tumorigenesis and progression of bladder cancer [137]. Deubiquitinating enzymes (DUBs), key enzymes that remove ubiquitin chains from their protein substrates, are involved in the regulation of different cellular processes including cell growth, & differentiation, and they can also play crucial roles in cancer development [138]. Ubiquitin C-terminal hydrolase-L5 (UCHL5), a member of the DUBs family of proteins that is overexpressed in bladder cancer, has been indicated to promote the growth and migration of bladder cancer cells by increasing MYC expression through the AKT/mTOR signaling pathway [139]. Similarly, SCL/TAL1 interrupting locus (STIL), an important checkpoint protein that is involved in the regulation of centriole duplication, is also highly expressed in bladder cancer, and it was reported that STIL enhanced the PI3K/AKT/mTOR pathway resulting in increased MYC expression, thereby promoting the development of bladder cancer [140].

Aberrant expression of noncoding RNAs (circRNAs, miRNAs, lncRNAs and PIWI interacting RNA (piRNA)) plays crucial roles in the initiation and progression of various cancers [141]. Circular RNAs (circRNAs) are single stranded covalently closed RNA molecules that play important roles in various biological functions as microRNA sponges & transcriptional regulators, and in carcinogenesis [142]. CircCDYL, which is derived from the exon 4 of CDYL through back splicing, inhibits bladder cancer cell growth by down regulating the protein level of MYC without altering mRNA level of MYC [143]. This downregulation of MYC protein may be through promoting its degradation but the detailed mechanism has not been reported [143]. Hsa_circ_0068307, a highly expressed circular RNA in UBC, has been shown to promote tumor growth in vitro and in vivo via sponging miR-147 and promoting the expression of MYC, which is a direct target of miR-147 [144]. Furthermore, an exonic circular RNA (circNR3C1) which is generated from NR3C1 gene, interacts with bromodomain-containing protein 4 (BRD4) causing dissociation of BRD4/MYC complex and preventing MYC function as a transcription factor to inhibit bladder cancer progression [145]. Many other noncoding RNAs also interact with MYC altering its expression, among which the microRNAs miR-451 and Let-7a, which directly target and down-regulate the expression levels of MYC, and SNHG18, a newly identified lncRNA that inhibits the proliferation of bladder cancer cells by decreasing MYC protein expression have been reported [146–148].

## Patterns of FGFR3 and c-MYC alterations in different stages and grades of urothelial bladder *cancer*

The pattern of distribution of *FGFR3* mutation and expression varies considerably throughout the different stages and grades of UBC. *FGFR3* mutations are more prevalent in tumors with low stage and low grade [80, 82, 86]. Billerey et al. reported FGFR3 mutations in 74% of pTa, 21% of pT1, 16% of pT2–4 tumors, and in 84% of G1, 55% of G2 and 7% G3 tumors [63]. In a meta-analysis which included 916 tumors, Neuzillet et al. has shown that the frequency of the *FGFR3* mutations decreased with increasing stage and grade, and reported *FGFR3* mutations in 65% of pTa, 30.2% of pT1, 11.5% of pT2-4 tumors, and in 69.8% of G1, 68% of G2 and 18.6% of G3 tumors [149]. It has also been demonstrated that FGFR3 expression follows the distribution of *FGFR3* mutations, with significantly higher levels of expression in low stage and grade bladder tumors than in invasive carcinomas [65, 80, 82, 84]. Few studies have examined *FGFR3* mutational status and expression levels in different parts of the same tumor sample or in paired samples of primary and metastatic tumors from the same patient. In a study reported by Tomlinson et al., out of 43 tumor blocks with both non-invasive and invasive regions, 18 had mutation in at least one region, including 9 with the same mutation in both regions and one with different mutations in the two regions, but 8 had mutation only in the non-invasive component out of which six were predicted to represent single tumors [80]. In the same study, 10 tumors had regions of both high and low FGFR3 expression, and a decrease in FGFR3 expression between the low and high stage regions was observed in seven tumors [80]. Additionally, Pouessel et al. found no discordance in FGFR status between the superficial and invasive parts of T1 tumors, but among 27 ≥ T2 TUR (Transurethral resection) samples, 8 had *FGFR3* mutation in the superficial part, with only 4 of them having *FGFR3* mutations in the invasive area [150]. This study has also revealed that FGFR3 status was similar in 201 paired cystectomies and metastatic lymph nodes (LNs) (10 of them harboring *FGFR3* mutations), and the authors suggested that the *FGFR3* mutation might be conserved in the invasive compartment and corresponding lymph node metastases if the mutated clone progresses to MIBC [150]**.** Furthermore, Turo et al., in a study of 106 matched pairs of primary tumors and LN metastases using IHC for evaluating FGFR3 expression, reported FGFR3 expression concordance between the primary tumors and metastatic LNs in 79 of them, with 15 patients exhibiting upregulated expression only the metastatic LNs and 12 patients only in the primary tumor [151].

The discordant *FGFR3* mutation status as suggested by the above studies where some tumors harboring *FGFR3* mutation early in the non-invasive stage convert to wild type *FGFR3* during tumor progression might reflect the importance of this pathway at an early stage of carcinogenesis. It has also been suggested that this loss of *FGFR3* mutation during tumor progression may potentially alleviate oncogene-induced upregulation of p16 and related cell cycle checkpoint genes allowing tumor progression [72]. In addition, studies have also indicated the possible role of loss of *CDKN2A* which encodes p16, in the progression of tumors with *FGFR3* mutation. It has been reported that the frequency of *CDKN2A* deletion is significantly higher in MIBCs with *FGFR3* mutation than in those with wildtype *FGFR3*, suggesting that MIBCs with *FGFR3* mutation and *CDKN2A* deletion might represent tumors that have progressed from *FGFR3* mutant NMIBC [55].

Alterations in expression levels of MYC and/or gene copy number changes are known to occur in UBC. However, data regarding correlation of these alteration with tumor grade and stage are conflicting. Some studies have reported that overexpression is associated with low stage and grade tumors while others have shown the lack of association with tumor stage or grade, or association with high grade and stage tumors. Fragkoulis et al. in a study involving 54 patients, MYC IHC positive staining was found in 89% (33/37) of NMIBCs, 53% (9/17) of MIBCs and in only one out of 5 lymph node metastases [19]. MYC IHC positive staining was also high in low grade tumors (93% (28/30)) vs 58% (14/24) in high grade tumors, and the authors reported that MYC IHC positive tumors are associated with low grade and non-muscle invasive tumors, while negative staining was associated with higher grade and higher stage disease [19]. Sauter et al. have also shown that MYC protein overexpression is associated with low grade and low stage tumors, and reported overexpression in 18/21(86%) of Ta, 9/13 (69%) of T1, 10/17(59%) of T2-4, 18/20 (90%) of G1, 13/18 (72%) of G2 and 5/12 (42%) of G3 tumors [152]. In this study, there was a tendency towards more frequent MYC overexpression in pTa/pT1 tumors than in T2–4 tumors, but without reaching statistical significance [152]. In contrast, Schmitz et al. reported MYC protein overexpression in 58% of Ta, 56% of T1 and 59% of MIBCs, with no correlation between overexpression and tumor grade or stage [134]. Additionally, Grapsa et al. in a study of 100 primary UBC samples reported lack of correlation between the protein expression levels of MYC and clinicopathological parameters including tumor stage or grade [153]. Few other studies have also failed to find any correlation between MYC mRNA expression levels and tumor stage or grade [133, 154]. On the other hand, significant correlation between high mRNA expression levels of MYC with higher histological grade and stage have been reported [155].

The reports regarding *MYC* gene copy number gains and amplifications appear relatively consistent, at least in those studies involving large samples. These studies have shown that *MYC* copy number increase was associated with advanced stage and high-grade tumors. Zaharieva et al. in a study of 2317 bladder cancer samples found *MYC* copy number gains in 10.5% of pTa, 15.8% of pT1, 21.4% of pT2–4, as well as in 5.7% of G1, 11.9% of G2 and 19.7% of G3 urothelial carcinomas [156]. *MYC* amplifications were also seen in 0.6% of pTa, 4% of pT1, 5.5% of pT2–4, as well as in 0.8% of G1, 1.7% of G2 and 4.7% of G3 urothelial carcinomas, with an overall amplification rate of 2.9% [156]. A recent report by Kluth et al., which also included a large number of samples (2052 UBCs), has revealed a low level *MYC* copy number gain (MYC/centromere 8 ratio ≥ 2 and ≤ 3) in 6.8% and high-level *MYC* amplifications (MYC/centromere 8 ratio > 3) in 3.3% of urothelial bladder carcinomas [157]. In this study, the rate of *MYC* copy number gain and amplification increased with increasing grade in pTa tumors and was highest in muscle invasive pT2-4 carcinomas where *MYC* copy number gain/amplification rate further increased from pT2 to pT4 [157]. Other studies have also confirmed these findings [152, 158], but in a study reported by Christoph et al. a statistically significant relationship between *MYC* gene copy number increase and tumor stage or grade was not found [133]. In the later study, only 4 Ta, 6 T1 and 37 T2-T4 tumors, with the far majority being high grade tumors were studied which might be responsible for the statistically insignificant relationship.

Some of the studies mentioned above have investigated both MYC overexpression and copy number gain/amplification in the same UBC samples [133, 152, 158]. In the first study (reference 133), MYC protein level was not investigated but a strong correlation between the copy number of the *MYC* gene and the level of its mRNA was described [133]. On the other hand, the other two studies (references 152 & 158) reported lack of association between MYC protein overexpression and low level *MYC* gene copy number gain or amplification [152, 158]. It was also described that most urothelial carcinomas with protein overexpression lack *MYC* amplification, and only some of the *MYC* amplified tumors overexpress MYC protein [152]. While gene amplification may underlie MYC protein overexpression in other cancer types, these studies suggested that *MYC* amplification is an uncommon cause of MYC protein overexpression in urothelial carcinoma providing evidence for the importance of other mechanisms of MYC dysregulation in UBC.

Studies investigating MYC dysregulation specifically in FGFR3 altered UBCs are rare. As pointed out earlier, upregulated MYC expression due to activated FGFR3 and a positive feedback loop between FGFR3 and MYC have been described. Despite this, studies reporting the correlation of MYC expression levels with tumor grade & stage seem inconsistent as described in this section, with some studies reporting association of MYC overexpression with low stage and low-grade tumors while others reporting the opposite or no association. In contrast, *FGFR3* mutations and overexpression are associated with low stage and low-grade tumors. This may point to the importance of other signaling pathways and mediators in MYC dysregulation in UBC. In fact, MYC is a convergence point of diverse signaling pathways, and complex interactions with different pathways and mediators (some of which were described in this review) could make the relationship between FGFR3 activation and MYC expression complex. Investigating such relations may provide further insight into the pathogenesis of UBC, and it may also have therapeutic implications as discussed next.

## Therapeutic implications

Early preclinical studies have provided evidence that FGFR3 is a valid therapeutic target in bladder cancer [13, 65, 95, 96]. In these studies, the antitumor activities of FGFR inhibitors have been observed in bladder cancer cells harboring *FGFR3* activating mutations, *FGFR3* fusions and overexpression [95, 96]. However, it is noted that the effects of FGFR inhibitors are predominantly cytostatic (cell cycle arrest in G1 or G0) rather than cytotoxic [61, 65, 95]. Furthermore, marked heterogeneity in their responses to FGFR inhibitors has been noted, and not all FGFR3 altered bladder cancer cells exhibit response. Studies in bladder cancer cell lines have showed that some FGFR3 altered cell lines are sensitive to FGFR inhibition while others are moderately sensitive or resistant. For example, some cell lines (UM-UC14, SW780, RT4, RT112 and UM-UC1) were found to be sensitive to FGFR inhibition while several other FGFR3 mutant cell lines (UM-UC6, UM-UC15, UMUC16, UM-UC17, 94-10, 97-18, J82) were not sensitive [61, 95, 96]. Little is known about the determinants of this differential sensitivity of FGFR3 altered bladder cancer cells to FGFR inhibition. A study has reported that EGFR activation rescues the MAPK pathway activity from FGFR inhibition induced transient downregulation of MAPK signaling, and this limits sensitivity to FGFR inhibition in partially FGFR3 dependent cell lines with *FGFR3* mutations or fusions [159]. Additionally, in some FGFR3 mutant cell lines with intrinsic resistance to FGFR inhibitors, EGFR dominates the downstream signaling through repression of mutant FGFR3 expression [159]. Although *FGFR3* mutation presumably initiated cancer development in these cancers, it has been suggested that at some point during tumor progression, EGFR signaling may have increased to a level where it repressed mutant *FGFR3* expression and dominated the downstream signaling [159]. Similarly, preclinical studies providing mechanistic basis for acquired resistance to FGFR inhibition suggested a dependency switch from FGFR to ERBB2/3 signaling pathway to compensate for FGFR inhibition and cause a rapid reactivation of the MAPK signaling [69, 160]. In fact, other mechanisms of resistance to FGFR inhibition through MET and AKT activation have also been reported in bladder cancer cell lines [161, 162].

A number of drugs targeting the FGFR signaling pathway have been developed including tyrosine kinase inhibitors, monoclonal antibodies and ligand traps, among which small molecule tyrosine kinase inhibitors are the most widely used therapeutic modality in cancer patients [163]. The pan-FGFR inhibitor erdafitinib has shown an overall response rate of 40% in bladder cancer patients whose tumors contained *FGFR3* point mutations or *FGFR2/3* fusions, leading to FDA approval of this drug in locally advanced or metastatic bladder cancer [164]. Other clinical trials have also demonstrated the benefits of other FGFR inhibitors in a subset of UBC patients, but with lower response rates [165]. The identification of the appropriate subset(s) of UBC patients who will benefit most from FGFR-directed therapy is a significant challenge as not all patients with FGFR3 mutated bladder tumors respond to FGFR inhibition, and there are also responders without FGFR3 alterations [166]. It has been suggested that *FGFR3* mutation which has a driver role may represent a better predictive biomarker for FGFR inhibitor therapies than upregulated expression of wildtype *FGFR3* which has a possible passenger role [82].

It has been suggested that MYC may function as a dominant downstream effector that mediates the antitumor effects of FGFR inhibition in FGFR addicted cancer cells. A study involving cancer cell lines harboring FGFR1, FGFR2 and FGFR3 alterations has described consistent reduction in MYC protein level upon FGFR inhibition in FGFR inhibitor-responsive cancer cells, while MYC level remained intact in FGFR inhibitor-nonresponsive cells despite obvious FGFR signaling inhibition [136]. In FGFR dependent lung cancer cells, induction of oxidative stress has been suggested to be the main mechanism responsible for lung cancer cell death following FGFR inhibition, and it has been reported that the reduction of MYC protein levels strictly determined the onset of oxidative stress [167]. Similarly, in multiple myeloma cells, the reduction of MYC levels induced by FGF/FGFR inhibition triggers oxidative stress, DNA damage and apoptosis [168]. Studies in FGFR3 dependent bladder cancer cells have also showed the key role of MYC in mediating growth inhibition due to FGFR blockade. In RT112 and MGH-U3 cell lines, a study has shown that either *FGFR3* or *MYC* knock down resulted in significantly lower cell viability, with no significant additive effect of simultaneous *FGFR3* and *MYC* knockdown [91]. Similarly, in FGFR3 mutated UMUC14 cell lines, *MYC* knockdown significantly suppressed cell survival and subsequent FGFR inhibition did not further inhibit cell survival, all suggesting that MYC inhibition alone was sufficient to recapitulate the proliferation inhibition caused by FGFR inhibitors [136].

These studies have also reported a stringent association between MYC protein level alterations and response to FGFR inhibition. In xenograft tumor models that harbor FGFR aberrations including FGFR3-driven UMUC14 model, intratumoral MYC protein level was profoundly decreased along with the strikingly inhibited tumor growth due to FGFR blockade in responders while in an FGFR-nonresponsive model, the intratumoral level of MYC following FGFR inhibitor treatment remained constant, regardless of the abolishment of FGFR signaling [136]. Strict correlation between FGF/FGFR blockade, MYC downregulation, oxidative stress and apoptosis have also been described in FGFR dependent lung cancer while neither MYC downregulation by FGF/FGFR inhibition nor oxidative stress or apoptosis was observed in FGFR independent xenografts [167]. Furthermore, initially FGFR inhibitor sensitive lung cancer cell lines were treated with gradually increasing concentrations of the FGFR inhibitor to acquire FGFR inhibitor resistance. Along with the development of acquired resistance, the initially reduced MYC protein levels due to FGFR inhibition were gradually restored in spite of the continued FGFR inhibition, and in the generated FGFR inhibitor resistant cell line, the MYC protein level remained intact upon FGFR inhibitor treatment suggesting the dissociation of MYC from FGFR signaling [136]. These data suggest that MYC may be used as an important biomarker in the treatment and follow up of patients with FGFR altered tumors being treated with FGFR inhibitors.

Studies on MYC expression in FGFR3 altered UBCs are rare and the frequency of MYC overexpression in this subgroup has not been reported. Despite this, studies on other cancers have indicated another possible therapeutic relevance of MYC expression levels in tumors with FGFR alterations. In FGFR1 amplified lung cancers, the levels of *MYC* gene expression predicted FGFR inhibitor sensitivity, and it has been suggested that high expression levels of MYC may be associated with FGFR inhibitor response [169]. MYC was found to be overexpressed in 40% of the FGFR1 amplified tumors which is consistent with the finding that only a proportion of FGFR1 amplified lung tumors respond to FGFR inhibition [169]. Other studies have also similarly reported that tumors co-expressing MYC and FGFR may be more sensitive to FGFR inhibitors [170]. In FGFR3 altered UBC, determination of MYC expression may aid in the selection of appropriate subjects who will benefit most from FGFR inhibitor therapy, but studies are needed to support this hypothesis.

Finally, the predictive value of FGFR3 alterations to platinum-based chemotherapy has not been explored extensively. However, in general, studies have indicated that the basal subtype of MIBC are chemotherapy sensitive, while the luminal subtype tumors, which are expected to have a relatively higher frequency of *FGFR3* mutations, fusions and overexpression, have lower response rates to chemotherapy [62, 171, 172]. Moreover, few studies have reported that FGFR3 alterations may be associated with lower responses to platinum-based chemotherapy [173, 174]. These studies have highlighted the importance of exploration of nonchemotherapeutic approaches which may be combined with conventional chemotherapy for improved efficacy. Preclinical studies have shown that MYC overexpression is associated with cisplatin resistance in UBC cell lines [175, 176]. In these studies, it has also been indicated that downregulation of MYC expression may enhance sensitivity of tumor cells to cisplatin [176–178]. Considering the stringent association between MYC protein level reduction and response to FGFR inhibition as discussed above, it would be possible to speculate that the intratumoral MYC protein levels could be low in UBC patients (with FGFR3 altered tumors) who respond to FGFR inhibitors. Taking this together with the potential role of MYC downregulation in enhancing cisplatin sensitivity as suggested by the above preclinical studies, it is possible that these group of UBC patients with response to FGFR inhibition may have a better response to cisplatin-based chemotherapy. In fact, enhanced cytotoxic effects of cisplatin due to FGFR inhibition have been reported in malignant pleural mesothelioma and small cell lung cancer cells, although the enhanced cisplatin activity was not attributed to MYC downregulation in these studies [179, 180].

## Conclusions

The role of FGFR3 in urothelial bladder cancer has been thoroughly studied and the central importance of FGFR3 activation in bladder cancer pathogenesis is well established. MYC is a key downstream effector of activated FGFR3 that mediates tumorigenesis in bladder cancer. Evidence linking FGFR alterations with MYC dysregulation and the potential therapeutic relevance has been shown in different cancers. It will be crucial to investigate MYC alterations in FGFR3 aberrant urothelial bladder cancer, as it may provide further insight into the pathogenesis of bladder cancer, and it could also have therapeutic implications.

## Data Availability

Not applicable.

## Abbreviations

AKT: Protein kinase B
APC: Adenomatous polyposis coli
AXIN: Axis inhibition protein
BAIAP2L1: Brain-specific angiogenesis inhibitor 1-associated protein 2-like 1
BAR: Bin-Amphiphysin-Rvs domain
bHLHLZ: Basic helix–loop–helix/leucine zipper
BRD4: Bromodomain-containing protein 4
CBL: Casitas B-lineage lymphoma proto-oncogene
CDK1: Cyclin dependent kinase 1
CDKN2A: Cyclin dependent kinase inhibitor 2A
circRNAs: Circular RNAs
CK: Casein kinase
DUBs: Deubiquitinating enzymes
DUSP: Dual specificity phosphatase
ERBB: Erythroblastic leukemia viral oncogene homologue
ERK: Extracellular signal regulated kinase
Fbw7: F-box and WD repeat domain-containing protein 7
FDA: US Food and Drug Administration
FGFR: Fibroblast growth factor receptor
FRS2: FGFR substrate 2
Gab1: Grb2-associated binding protein 1
Grb2: Growth factor receptor-bound 2
GSK: Glycogen synthase kinase
HIF: Hypoxia-inducible factor
HSPGs: Heparan sulfate proteoglycans
ICI: Immune check point inhibitor
Ig: Immunoglobulin
IHC: Immunohistochemistry
KLPH: Klotho-LPH related protein
lncRNAs: Long noncoding RNAs
LNs: Lymph nodes
MAPK: Mitogen activated protein kinase
MB: MYC or M boxes
MIBC: Muscle invasive bladder cancer
miRNAs: MicroRNAs
MKP: MAPK phosphatases
mRNA: Messenger RNA
NEMO: NF-kB essential modulator
NMe: NOTCH MYC enhancer
NMIBC: Non-muscle invasive bladder cancer
PI3K: Phosphatidyl inositol 3 kinase
piRNA: PIWI interacting RNA
PLCγ: Phospholipase C gamma
POLD: DNA polymerase δ
PP2A: Protein phosphatase 2A
PTB: Phosphotyrosine-binding domain
RB: Retinoblastoma
RSK: Ribosomal S6 kinase
RTKs: Receptor tyrosine kinases
SCF: Skp1-Cullin-F-box-protein complex
SEF: Similar expression to *fgf* genes
SH: Src homology
siRNAs: Small interfering RNA
Skp2: S-phase Kinase-Associated Protein 2
SNPs: Single nucleotide polymorphisms
SOS: Son of sevenless
Spry: Sprouty
STAT: Signal transducers and activators of transcription
STIL: SCL/TAL1 interrupting locus
TACC3: Transforming acid coiled-coil containing protein 3
TAD: Transactivation domain
T-ALL: T-cell acute lymphoblastic leukemia
TGF-β: Transforming growth factor-β
TUR: Transurethral resection
TURBT: Transurethral resection of bladder tumors
UBC: Urothelial bladder carcinoma
UCHL5: Ubiquitin C-terminal hydrolase-L5
